# Effects of *Arum dioscoridis* Extract on Hepatic Toxicity Caused by Thioacetamide in Rats

**DOI:** 10.5152/tjg.2023.211051

**Published:** 2023-03-01

**Authors:** Murat Fatih Sökmen, Murat İspiroğlu, Kadir Gişi, Abdulkadir Yasir Bahar, Ergül Belge Kurutaş, Bülent Kantarçeken

**Affiliations:** 1 Department of Gastroenterology, Kahramanmaraş Sütçü İmam University Faculty of Medicine, Kahramanmaraş, Turkey; 2 Department of Internal Medicine, Kahramanmaraş Sütçü İmam University Faculty of Medicine, Kahramanmaraş, Turkey; 3 Department of Pathology, Kahramanmaraş Sütçü İmam University Faculty of Medicine, Kahramanmaraş, Turkey; 4 Department of Biochemistry, Kahramanmaraş Sütçü İmam University Faculty of Medicine, Kahramanmaraş, Turkey

**Keywords:** *Antioxidant effect*, Arum dioscoridis, hepatotoxicity, thioacetamide

## Abstract

**Background:**

The aim of this study was to investigate the prophylactic and therapeutic effects of *Arum dioscoridis* (tirsik) plant extract against thioacetamide-induced experimental liver toxicity.

**Method:**

s**: **In this study, 35 male Wistar–Albino rats, of 12-14 weeks old, weighing between 200 and 270 g, were used. Rats were divided into 5 groups of 7 each. The first group was determined as the control group, the second group as the hepatotoxicity group, the third group as the prophylaxis group, the fourth group as the intraperitoneal treatment group, and the fifth group as the oral treatment group. Hepatotoxicity was achieved with a single intraperitoneal dose of 350 mg/kg of thioacetamide (TAA). On the seventh day, the rats were sacrificed under general anesthesia. Their blood was taken and liver enzymes were studied. Malondialdehyde (MDA), glutathyon peroxidase (GPx), catalase (CAT), superoxit dismutase (SOD) enzymes were studied from liver tissues. In addition, liver tissues were evaluated histopathologically.

**Results:**

With *Arum dioscoridis* treatment and prophylaxis, improvements in all parameters and increases in tissue antioxidant levels were detected.

**Conclusion:**

It was determined that *Arum dioscoridis* plant extract has prophylactic and therapeutic effects on liver toxicity. In cases of acute liver injury and hepatotoxicity, we suggest the potential application of *Arum dioscoridis* for effective and inexpensive treatment.

**Main Points**

- *Arum dioscoridis* (Tirşik) is a plant with strong antioxidant and antibacterial effects and is mostly consumed as a soup in our region.
- This is the first study to examine the hepatoprotective properties of *Arum dioscoridis*.
- Most of the antioxidant ability of arum dioscoridis is due to vitexin and ferulic acid content.
- It was observed that *Arum diascoridis* has both therapeutic and prophylactic benefits in liver toxicity.
- We observed its efficacy in serum laboratory values and liver biopsy examination.

## Introduction

Cirrhosis is a chronic disease associated with many liver diseases and conditions such as hepatitis and chronic alcoholism. Studies have shown that free radicals play a role in the process of progression to fibrosis, cirrhosis, and liver cancer following inflammatory conditions in liver diseases. Oxidative stress occurs when the balance between free radicals and antioxidants is in favor of free radicals.^1^ Oxidative damage occurs in macromolecules like DNA, proteins, and lipids subsequent to the increased oxidative stress. DNA bases which undergo oxidation and lipid peroxidation play an important role in liver damage and carcinogenesis.^2,3^

There are antioxidant defense systems in the human body which inhibit the free radicals produced due to physiologic or abnormal conditions.^4^ Examples of these antioxidants are vitamins A, C, E, beta carotene, melatonin, nicotinamide adenine dinucleotide phosphate (NADPH), adenosine, coenzyme Q-10, urate, ubiquinol, poliphenols, flavonoids, phytoestrogens, amino acids, nitric oxide, glutathione, glutathione peroxidase, catalase, superoxide dismutase, thioredoxine reductase, and nitric oxide synthetase.^5,6^ Antioxidants are compounds which prevent lipid peroxidation, DNA mutation, and cross-linking of proteins. Free radicals are also responsible for cancer and most antioxidant substances may stop cancer in the first step and prevent its progression to tumors.^7,8^ TAA is a toxic substance with thiono-sulfur content, which has necrogenic and carcinogenic effects, and was used for hepatoxicity in our study.^9,10^

Currently, natural antioxidant compounds are used in the treatment of liver disorders besides medical therapy, surgical treatment, and radiotherapy. Flavonoids are also among these compounds. Currently, flavonoids with known antioxidant properties gain increasing importance due to the increased incidence of, especially, non-alcoholic fatty liver disease.^11^

*Arum dioscoridis* (AD) plant is known as “tirşik = sour iron” among the population in the Eastern Mediterranean region of our country, particularly in the Kahramanmaraş/Andırın district.^12^ It is mostly consumed as a soup. The plant mainly contains carbohydrates, proteins, free amino acids, and ascorbic acid.^13^ As antioxidants, they have high activity, especially regarding catalase and SOD enzymes.^12^

We have not yet encountered a research in the literature studying the effects of AD plant extract on the liver. In our study, possible protective and therapeutic effects of AD on the liver toxicity induced by thioacetamide were analyzed.

## Materials and Methods

In our study, AD (tirşik) plant collected from the countyside of Andırın county of Kahramanmaraş province was cleaned at the laboratory and the species of the plant was identified with the aid of the relevant literature.^14^ The leaves were dried at room conditions. Dried leaves were made ready for use in 5-g cartridges to be extracted in the soxhlet device. The prepared cartridge was extracted in 100 mL solvent for 6 hours and methanol was used as the solvent for extraction.^15^ The extract obtained was evaporated in a rotary evaporator until 2 mL remained. Evaporation process was adjusted according to the boiling point temperature of the solvent. The prepared extract sample was preserved at +4°C until it is used.^16^ The analysis performed revealed a humidity content of 38% ± 5% of the plant. The ratio of the extract obtained from the plant in the extraction with methanol was found to be 42.5%. The extract obtained was dissolved in pure water with 10% methanol (50 cc methanol+450 cc pure water) to be used in the experiment and a 500 cc homogeneous solution was obtained. Dosing was made from this homogeneous solution at a dose of 2 mL/kg/day and was administered to the rats for the experiment.

At the beginning of the experiment, the toxic substance and plant extract were administered according to the weights of the rats. At the end of the experiment, 1 rat in the fifth group died. Intracardiac blood was taken from the remaining rats for biochemical investigations just before sacrificing them on the seventh day with ketamine-induced general anesthesia. After the rats were sacrificed, liver tissue samples were taken for histopathologic examination and tissue biochemistry. The blood samples were centrifuged for 10 minutes at 4000 revolutions. Later, the serum part was transferred into eppendorf tubes and were kept at −80°C until analysis. Sections of 0.5 cm thickness were taken from the liver tissues and were fixed in 10% formalin and buried in paraffin blocks; 4 μm-thick sections were taken from the blocks, stained with hematoxylene–eosin (HE) for histopathologic examination, and evaluated under light microscope. Some liver tissue was preserved at −80°C until analysis for biochemical studies.

Based on histopathologic findings, they were classified into 5 grades as grade 0 (no pathology), grade 1 (single cell necrosis and mild hepatocyte damage, no centrilobular necrosis), grade 2 (single cell necrosis and intense hepatocyte damage, no centrilobular necrosis), grade 3 (mild centrilobular necrosis), grade 4 (marked centrilobular necrosis), and grade 5 (centrilobular and midzonal necrosis).^17^

### Experimental Animals

In total, 35 Wistar–Albino male rats of 12‐14 weeks, weighing between 200 and 270 g, were used in the study. The rats were kept at room conditions (22 ± 2°C) and fed with standard pellet rat feed. The rats were divided into 5 groups of 7 rats in each group. The study lasted for 7 days. Nutrition and medicine applications of rat groups were carried out in our institution’s animal laboratory by an authorized personel.

Group 1 (control group, n = 7): normal physical conditions were followed.

Group 2 (group undergoing liver toxicity, n = 7): normal physical conditions were followed. On the sixth day, a single dose of TAA of 350 mg/kg/mL was given via intraperitoneal (i.p.) route.

Group 3 (group undergoing liver toxicity following prophylactic AD, n = 7): following 2 mL/kg/day of peroral (p.o.) AD for 5 days, a single dose of 350 mg/kg/mL of TAA was given via i.p. route on the sixth day.

Group 4 (group given i.p. AD following liver toxicity, n = 7); on the first day, a single dose of i.p. TAA of 350 mg/kg/mL was given, and 24 hours later, i.p. AD was given at 2 mL/kg/day for 5 days.

Group 5 (group given p.o. AD following liver toxicity, n = 7): on the first day, a single dose of i.p. TAA of 350 mg/kg/mL was given, and 24 hours later, p.o. AD was given at 2 mL/kg/day for 5 days.

### Statistical Analysis

Normality of the data distribution was analyzed using the Shapiro–Wilk test. Comparisons of 5 groups were analzyed using the Kruskal–Wallis H test for variables with abnormal distribution. Dunn–Sidak test was applied for the post hoc test. One-way analysis of variance test was used for the comparison of 5 groups for variables with abnormal distribution. Dunnett test, Tukey test, and Tamhane T2 tests were performed for the post hoc test. *P* <.05 was considered statistically significant. Statistical parameters were considered for median (min-max) values. Data were evaluated with the International Business Machines (IBM) Statistical Package for the Social Sciences version 22 (IBM Corp.; Armonk, NY, USA).

## Results

Alkaline phosphatase (ALP), alanine aminotransferase (ALT), aspartate aminotransferase (AST), gamma-glutamyl transferase (GGT) levels were significantly higher in all groups who were administered TAA compared to the control group (*P*  < .001). Increase in AST, ALT, ALP, and GGT levels in the groups receiving therapy (group 4 and group 5) was noted to be less than those in the group not receiving therapy (group 2), but this was not statistically significant (*P*  > .05).

However, ALT level was significantly lower in the group receiving prophylactic therapy (group 3) compared to the group not receiving therapy (group 2) (*P*  < .001) (Table 1).

Compared with the control group, there was a significant increase in total serum bilirubin levels in all the groups except for the group receiving prophylactic therapy (*P*  < .001). On the other hand, total serum bilirubin levels of the prophylaxis group were almost the same as those of the control group and were significantly lower than the toxicity group (group 2) (*P*  < .001) (Table 1).

Serum albumin levels were significantly lower in all groups with induced toxicity compared to the control group (*P*  < .001). Albumin level was significantly higher in all the groups receiving therapy than the group not receiving therapy (group 2) (*P*  < .001) When the treatment groups were compared, the group with the significantly highest albumin level was the group receiving prophylactic therapy (group 3) (*P*  < .05). On the other hand, albumin level was significantly higher in the group receiving i.p. therapy (group 4) than the group receiving oral therapy (group 5) (*P*  < .001) (Table 1).

**Figure 1.**
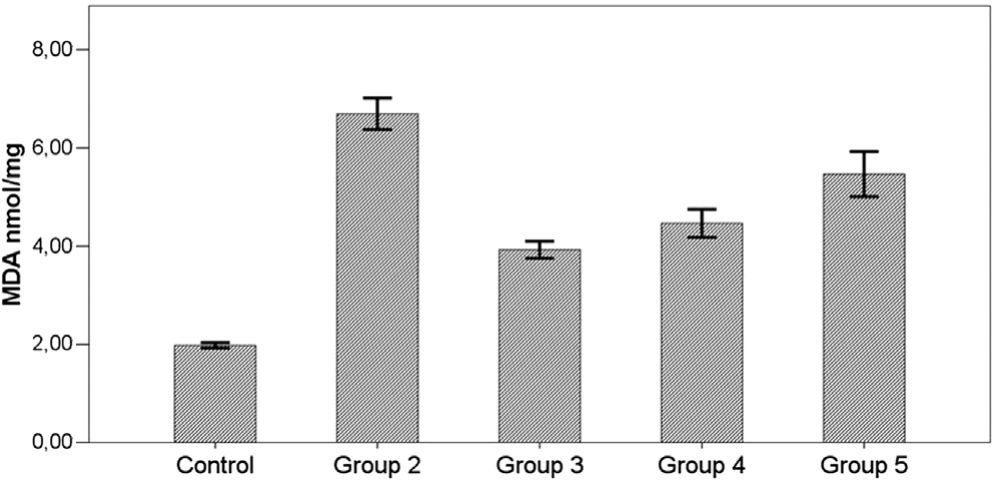
Effects of *Arum dioscoridis* therapy on malondialdehyde levels. Control, control group; group 2, toxicity group; group 3, prophylactic therapy group; group 4, intraperitoneal therapy group; group 5, oral therapy group. Control with groups 2-3-4-5, *P*  < .001; Group 2 with 3-4-5, *P*  < .001.

In all groups with induced toxicity, MDA level was signifivantly higher than the control group (*P*  < .001). In the groups receiving therapy (group 3, group 4, and group 5), MDA level was lower than the group not receiving therapy (group 2) (*P*  < .001), but the difference in the group receiving oral therapy (group 5) was not at a significant level (*P*  > .05) (Figure 1) (Table 2).

**Figure 2.**
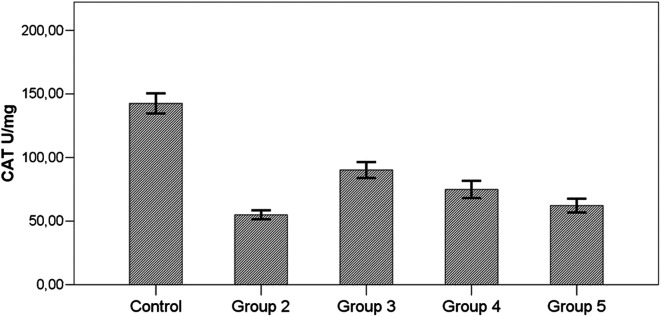
Effects of *Arum dioscoridis* therapy on catalase levels. Control, control group; group 2, toxicity group; group 3, prophylactic therapy group; group 4, intraperitoneal therapy group; group 5, oral therapy group. Control with groups 2-3-4-5, *P*  < .001; Group 2 with group 3, *P*  < .001; Group 2 with groups 4-5, *P*  > .05; Group 4 with group 5, *P*  > .05.

In all groups with induced toxicity, catalase level was significantly lower than the control group (*P*  < .001). In the groups receiving therapy (group 3, group 4, group 5), catalase level was higher than the group not receiving therapy (group 2). However, this increase in catalase level was significant only in the group receiving prophylactic therapy (*P*  < .001). Catalase levels were similar between the other two treatment groups (groups receiving i.p. and p.o) (*P*  > .05) (Figure 2) (Table 2).

**Figure 3.**
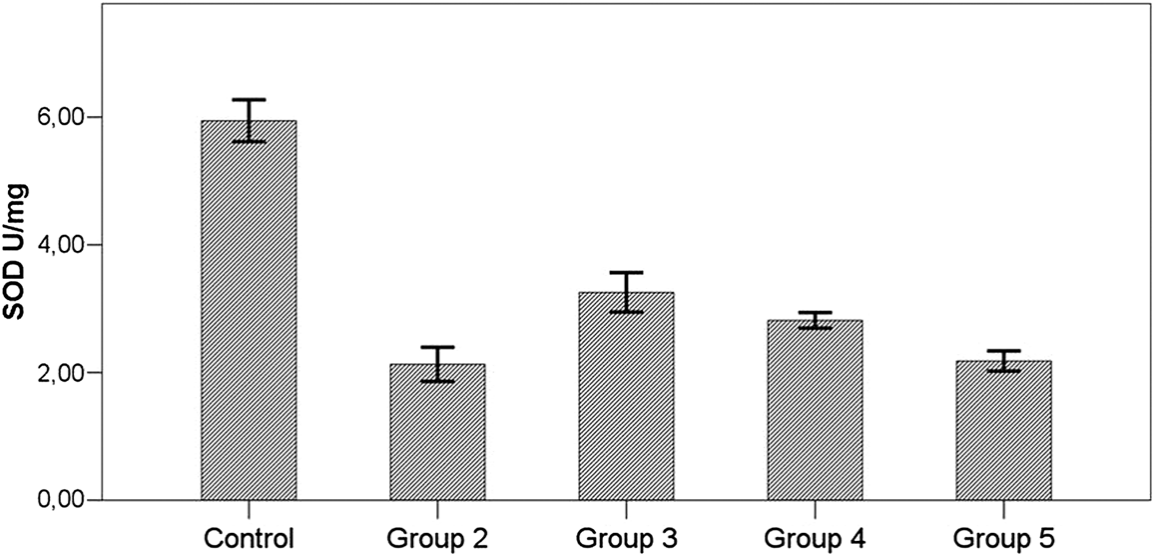
Effects of *Arum dioscoridis* therapy on superoxide dismutase levels. Control, control group; group 2, toxicity group; group 3, prophylactic therapy group; group 4, intraperitoneal therapy group; group 5, oral therapy group. Control with groups 2-3-4-5, *P*  < .001; Group 2 with group 3, *P*  < .001; Group 2 with groups 4-5, *P*  > .05; Group 4 with group 5, *P*  > .05.

SOD level in all the toxicity groups was significantly lower than the control group (*P*  < .001). SOD level was also higher in the groups receiving therapy (group 3, group 4, group 5) than that in the group not receiving therapy, but this difference was statistically significant in the group receiving prophylaxis (*P*  < .001) (Figure 3) (Table 2).

When i.p. treatment group was compared with the p.o. treatment group, SOD levels were higher in the i.p. treatment group, but the difference was not statistically significant (*P*  > .05) (Table 2).

**Figure 4.**
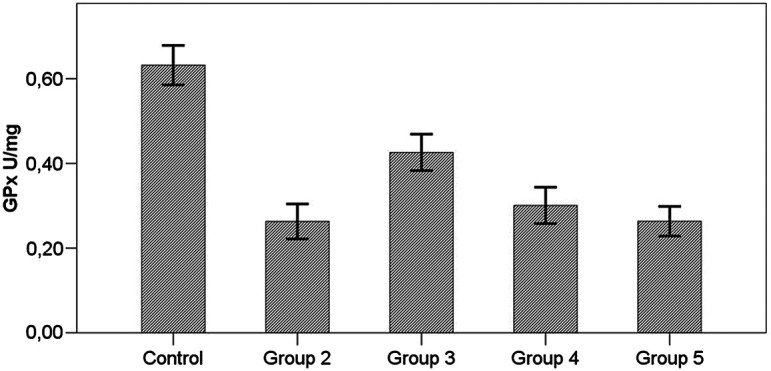
Effets of *Arum dioscoridis* therapy on glutathione peroxidase levels. Control, control group; group 2, toxicity group; group 3, prophylactic therapy group; group 4, intraperitoneal therapy group; group 5, oral therapy group. Control with groups 2-3-4-5, *P*  < .001; Group 2 with groups 3-4-5, *P*  > .05; Group 4 with group 5, *P*  > .05.

In all the groups with induced toxicity, GPx level was significantly lower than the control group (*P*  < .001). In the groups receiving treatment, GPx level was higher than the group not receiving treatment (group 2), but this was not statistically significant (*P*  > .05). GPx levels were not significantly different among the groups receiving treatment (*P*  > .05) (Figure 4) (Table 2).

**Figure 5.**
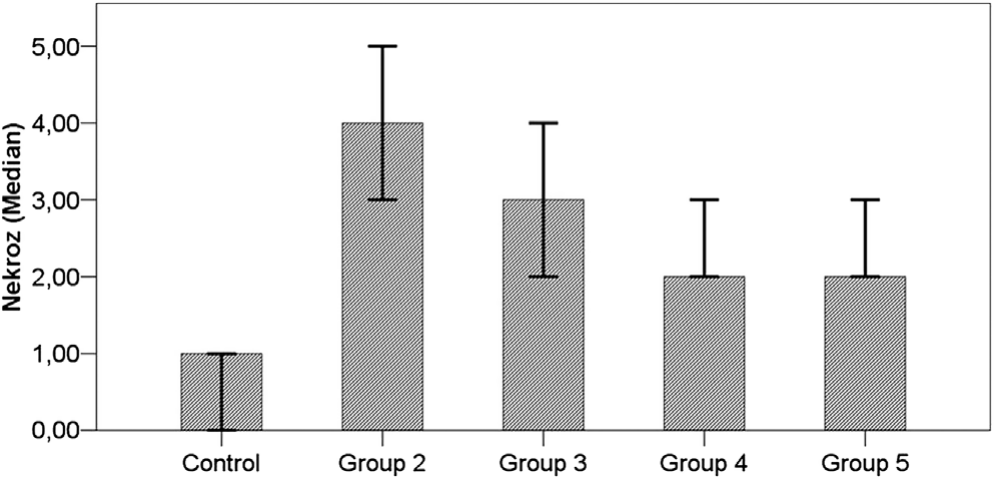
Results of histopathologic evaluation of *Arum dioscoridis* therapy. Control, control group; group 2, toxicity group; group 3, prophylactic therapy group; group 4, intraperitoneal therapy group; group 5, oral therapy group. Control with group 2, *P*  < .001; Control with group 3, *P*  < .001; Group 2 with group 3, *P*  > .05; Group 2 with groups 4-5, *P*  > .05.

**Figure 6.**
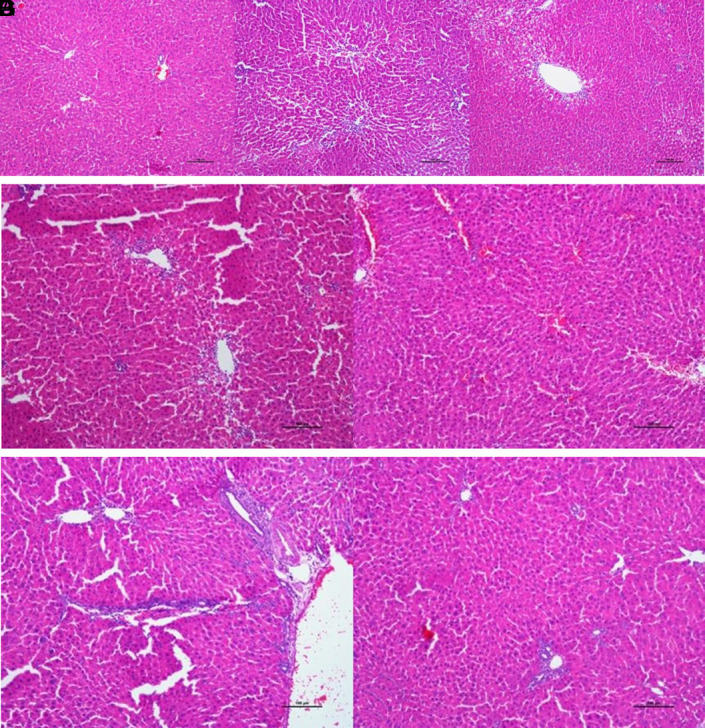
*(A) Control group:* appearance of a normal liver tissue (H&E ×100). *(B-C) Group with induced thioacetamide toxicity:* areas of marked centrilobular and midzonal necrosis (H&E ×100). (D) Group receiving prophylaxis: development of mild centrilobular necrosis and single cell necrosis, there is no severe hepatocyte damage (H&E ×100). (E) Group receiving intraperitoneal therapy: single cell necroses and hepatocyte damage are observed. While there is no sign of centrilobular necrosis, there is also regenerative atypia (H&E ×100). (F-G) Group receiving oral therapy: single cell necroses and hepatocyte damage are observed. While there is no sign of centrilobular necrosis, there is also regenerative atypia (H&E ×100).

In our study, cellular necrosis, hepatocyte damage and degree of centrilobular necrosis in liver tissue were evaluated in histopathologic evaluation. In the group with induced hepatotoxicity, marked centrilobular necrosis areas compared with the control group were noted (*P*  < .001) (Figure 5, 6A-C). In the prophylaxis group, areas of mild centrilobular necrosis were noted, and while the necrosis was statistically significant compared with the control group (*P*  < .001), its difference from the necrosis developing in the hepatotoxicity group was not statistically significant although it was milder (*P*  > .05). In i.p. and oral treatment groups, areas of regeneration and regeneration atypias were present in microscopic images. Diffuse hepatocyte damage and areas of marked centrilobular necrosis observed in the hepatotoxicity group (group 2) were replaced by areas of regenerative atypia and single cell necroses (group 4 and group 5), and areas of centrilobular necrosis were not noted. However, a statistical significance was not found (*P*  > .05) (Figure 6D-G) (Table 3).

## Discussion

Liver damage occurs related to numerous causes including viral hepatitides, alcohol use, Wilson’s disease, and consumption of some drugs continuously or at excessive doses. In this study, we induced hepatotoxicity with TAA which is used in many experimental models.

In a study performed by Koblihova et al^18^ the optimum TAA dose for the induction of liver toxicity in rats was found to be single dose of 350 mg/kg given via the i.p. route. Similarly, we also administered TAA at a single dose of 350 mg/kg and observed liver damage 1 day later.

*Arum dioscoridis*, which is an angiosperm in the monocot group, is a toxic plant that even animals refuse to eat. It is claimed that the liquid of this plant is effective against ear ache and the cancer.^19^ *Arum dioscoridis* has been observed in the studies that optimal antioxidant effect is obtained when 1 mg/mL methanol or ethanol extract is used for dissolving AD extract.^13^ In studies performed using high performance liquid chromatography analysis, antioxidant activity of AD was explained with its higher antioxidant phenolic compound (vitexin, ferulic acid, naringin, eriodictyol, etc.). In addition, it has been shown that methanolic extract of AD displays more potent antioxidant activity associated with polar phenolic compounds including vitexin and ferulic acid (Table 4).^20^

Therefore, we also used methanol as a solvent in our study, but there is no optimal dose level of AD extract recommended for animals in the literature. Therefore, taking into account the gastric capacity of rats, AD was administered at a dose of 2 mL/kg/day.

When the control group and other groups given TAA (groups 2-5) were compared, plasma AST, ALT, ALP, GGT, total bilirubin, albumin levels, tissue oxidative damage markers, and histopathological findings fully supported liver damage, and AST, ALT, total bilirubin levels were lower and albumin levels were higher in all the rats receiving AD (groups 3-5) compared to the group not receiving AD (group 2). However, among the groups receiving AD (group 3-5), the decrease in ALT and total bilirubin levels and increase in albumin levels were statistically significant only in the group taking prophylactic AD (*P*  < .05).

Even in the group given prophylactic AD (group 3), serum total bilirubin levels were almost at the same level as the control group. However, the decrease in total bilirubin levels in the treatment groups (groups 4 and 5) was not statistically different from the hepatotoxic group (group 2) (*P*  > .05). In the data obtained, serum albumin level in the prophylaxis group (group 3) was found to be significantly higher (*P*  < .001) than in the treatment groups (groups 4 and 5). When the serum albumin levels of the treatment groups were compared, it was found that the i.p. AD group (group 4) was significantly higher than the oral AD group (group 5) (*P*  < .001). However, when the bile duct-related enzyme (ALP and GGT) levels were examined, it was observed that there was no significant difference between the toxicity group (group 2) in both the prophylactic (group 3) and treatment groups (groups 4 and 5). This made us think that the antioxidant effect of AD on the biliary tract may be less or delayed.

In our study, antioxidant (SOD, GPx, and catalase) levels were significantly higher and MDA levels were significantly lower in all AD groups compared to the hepatotoxicity group (group 2).

MDA levels increased in all groups given TAA (groups 2-5) compared with the control group (*P*  < .001). This increase in the MDA level in liver tissue proves that TAA leads to lipid peroxidation and consequently to oxidative stress. Information obtained in our study about lipid peroxidation was similar to previously performed studies.^21-23^

MDA levels in the groups receiving prophylactic (group 3) and i.p. AD treatment group (group 4) were significantly less than those in the hepatotoxicity group (group 2) (*P*  < .001). MDA levels in the oral therapy group (group 5) were also less than those in the hepatotoxic group, but this difference was not statistically significant (*P*  > .05).

In our study, it was concluded that AD plant extract had a peroxidation reducing effect, especially when used prophylactically and intraperitoneally in the case of increased lipid peroxidation caused by TAA. However, although the MDA level in oral treatment AD group (group 5) was not significantly lower than hepatotoxic group (group 2), it was thought to be worth considering.

In rats with liver damage, increase in cellular damage occurs as a result of decrease in the activities of antioxidans (SOD, CAT, GPx) and increase in free radical levels.^24^ In the study by K.Sinan Dayısoylu^12^, it has been reported that AD has antioxidant properties and its activity values are at a rather good level just like some other plants with established antioxidant properties (for instance, common nettle) and has high activity, especially regarding catalase and SOD enzymes.

It was observed in our study that SOD, CAT, and GPx levels were significantly decreased in all groups receiving TAA (groups 2-5) compared with the control group (*P*  < .001). When SOD and catalase levels of the prophylactic group (group 3) were compared with those of the hepatotoxicity group (group 2), they were significantly higher (*P*  < .001). Although SOD and catalase levels of i.p. (group 4) and oral treatment group (group 5) were higher than those of the hepatotoxicity group, the difference was not statistically significant (*P*  > .05). When i.p. (group 4) and oral treatment groups (group 5) were compared, SOD and catalase levels were higher in the i.p. treatment group (group 4). But this difference was not statistically significant (*P*  > .05).

Van Remmen et al^25^ have shown a decrease in the reduced glutathione (GSH)/oxidized glutathione (GSSG) ratio and increase in lipid peroxidation with decreased catalase and GPx expression as a result of TAA administration in rat hepatocyte cultures.^26^

In our study, GpX levels were not significantly different from the hepatotoxic group (group 2) in both the prophylactic (group 3) and treatment groups (groups 4 and 5). In fact, oral treatment AD group (group 5) was also found to be similar to the hepatotoxicity group. This shows that SOD and catalase effects of AD are more prominent as stated in the literature,^12^ but the GpX activity is also lower.

These findings suggest that although AD plant extract is effective, especially in prophylactic use, i.p. administration is superior to oral administration in the treatment group.

Our histopathologic examination included the evaluation of cellular necrosis, hepatocyte damage, degrees of centrilobular necrosis in the liver tissue, and effects of AD prophylaxis and therapy on hepatocyte microarchitecture. Similar to the literature, in our study, significant hydropic degenerations and inflammation in the portal area and centrilobular necrosis were observed in the hepatotoxicity group compared to the control group (*P*  <.001).^27^

When compared with the hepatoxicity group (group 2), it was observed that the distinct centrilobular and midzonal necrosis areas were replaced by individual cell necrosis and mild hepatocyte damage in all groups treated with AD (groups 3-5).

Although this histopathological improvement was more significant in the prophylactic AD group (group 3), it was not statistically significant (*P*  >  0.05).

Although these results were not statistically significant (*P*  > .05), development of regenerative atypia areas is important regarding treatability and suggests that prolongation of treatment times can lead to full recovery.

When tissue antioxidant and serum biochemistry values were examined, it was thought that the reason for the lower treatment efficacy of AD than its prophylactic efficacy might be due to the short duration of AD application. However, if AD had been administered intraperitoneally to the prophylactic group (group 3), perhaps the hepatoprotective advantage would have been significant.

In addition, we could not find a reference for AD administration dose because there were no similar studies in the literature. *Arum dioscoridis* was administered both i.p. and p.o at 2 mL/kg/day because its toxic dose range is not clearly known. If a higher oral dose had been given, maybe the difference between i.p. and oral efficacy would not have been so great.

## Conclusion

This is the first study that examines the effects of AD on liver toxicity induced by thioacetamide in an experimental animal model.

According to the results of biochemical parameters, results of oxidation markers and histopathologic obtained, it was shown that AD has both preventive efficacy in hepatotoxicity (prophylactic significance) and therapeutic efficacy toward developed hepatotoxicity. Our study forms the basis of future research about the use of AD in the prophylaxis and treatment of acute liver damage and hepatotoxicity. We think that studies investigating treatment and prophylaxis options with different solvents and at different doses with longer toxicity models could be useful.

## Figures and Tables

**Table 1.** Results of Blood Biochemistry Parameters

	Control Group 1 (n = 7)	Hepatotoxicity Group 2 (n = 7)	Prophylaxis Group 3 (n = 7)	Intraperitoneal Therapy Group 4 (n = 7)	Oral Therapy Group 5 (n = 6)	*P*
AST (U/L)	13.06 ± 2.63^b,c,d,e^	33.91 ± 6.60^a^	27.11 ± 7.65^a^	30.61 ± 4.33^a^	34.66 ± 4.95^a^	< .001^*^
ALT (U/L)	16.53 ± 2.56^b,c,d,e^	38.57 ± 8.58^a,c^	25.07 ± 6.50^a,b^	30.65 ± 5.46^a^	32.39 ± 5.48^a^	< .001^*^
ALP (ng/mL)	4.55 ± 0.58^b,c,d,e^	21.17 ± 3.19^a^	16.61 ± 3.36^a^	19.18 ± 1.45^a^	21.13 ± 2.11^a^	< .001^*^
GGT (U/L)	9.61 ± 0.91^b,c,d,e^	22.90 ± 5.30^a^	17.81 ± 2.41^a^	22.32 ± 3.61^a^	22.75 ± 2.91^a^	< .001^*^
Total bilirubin (umol/L)	2.21 ± 0.42^b,d,e^	3.35 ± 0.81^a.c^	2.15 ± 0.45^b,d^	3.15 ± 0.53^a,c^	3.03 ± 0.39^a^	< .001^*^
Albumin (pg/mL)	70.06 ± 5.36^b,c,d,e^	42.30 ± 3.32^a,c,d,e^	58.61 ± 3.34^a,b,e^	59.00 ± 2.04^a,b,e^	51.86 ± 5.55^a,b,c,d^	< .001^*^

^*^Significantly different; ^a^Compared with the control group; ^b^Compared with group 2; ^c^Compared with group 3; ^d^Compared with group 4; ^e^Compared with group 5.

Note: Data are given as mean±standard deviation.

*P <.001 is statistically significant.

**Table 2.** Results of Oxidative Stress and Antioxidant Parameters

	Control Group 1 (n = 7)	Hepatotoxicity Group 2 (n = 7)	Prophylaxis Group 3 (n = 7)	Intraperitoneal Therapy Group 4 (n = 7)	Oral Therapy Group 5 (n = 6)	*P*
MDA (nmol/mg protein)	1.98 ± 0.15^b,c,d,e^	6.69 ± 0.84^a,c,d^	3.92 ± 0.46^a,b^	4.47 ± 0.76^a,b^	5.47 ± 1.12^a^	< .001^*^
CAT (U/mg protein)	142.53 ± 20.91^b,c,d,e^	54.98 ± 9.29^a,c^	90.18 ± 16.60^a,b,e^	74.91 ± 17.89^a^	62.24 ± 13.29^a,c^	< .001^*^
SOD (U/mg protein)	5.94 ± 0.87^b,c,d,e^	2.13 ± 0.71^a,c^	3.26 ± 0.82^a,b^	2.82 ± 0.32^a^	2.18 ± 0.39^a^	< .001^*^
GPx (U/mg protein)	0.63 ± 0.12^b,c,d,e^	0.26 ± 0.11^a^	0.43 ± 0.11^a^	0.30 ± 0.11^a^	0.26 ± 0.09^a^	< .001^*^

^*^Statistical significance; ^a^Difference from control group significant; ^b^Difference from group 2 significant; ^c^Difference from group 3 significant; ^d^Difference from group 4 significant; ^e^Difference from group 5 significant. Data are given as mean ± standard deviation.

*P <.001 is statistically significant.

**Table 3.** Results of Histopathologic Evaluation

	Control Group 1 (n = 7)	Hepatotoxicity Group 2 (n = 7)	Prophylaxis Group 3 (n = 7)	Intraperitoneal Therapy Group 4 (n = 7)	Oral Therapy Group 5 (n = 6)	*P*
Necrosis median (min-max)	1.00 (0.00-1.00)^b,c^	4.00 (3.00-5.00)^a^	3.00 (2.00-4.00)^a^	2.00 (2.00-3.00)	2.00 (2.00-3.00)	< .001^*^

*Statistical significance; ^a^Difference from control group significant; ^b^Difference from group 2 significant; ^c^Difference from group 3 significant; ^d^Difference from group 4 significant; ^e^Difference from group 5 significant.

**Table 4.** High Performance Liquid Chromatography Analysis of *Arum dioscoridis* Plant Extracts

Content	Methanolic Extract	Acetone Extract	Hexane Extract
Gallic acid	-	-	-
Catechin hydrate	-	-	-
Caffeic acid	-	-	-
Epicatechin	-	-	-
p-Coumaric acid	5.4 ± 0.8	5.6 ± 0.4	-
Ferulic acid	325.5 ± 2.5	255.9±1.9	-
**Vitexin**	**1125.0 ± 13.4**	**-**	**23.4 ± 2.4**
Rutin	-	-	-
Naringin	98.4 ± 5.5	19.4 ± 3.2	-
Hesperidin	-	-	-
Rosmarinic acid	-	-	-
Eriodictyol	43.7 ± 3.1	25.0 ± 4.2	5.2 ± 0.7
Quercetin	-	-	-
Naringenin	-	-	-
Carvacrol	-	-	-
Σ Total	1598.0	305.9	34.2

Vitexin level was observed at the highest level especially in methanolic extract of arum dioscoridis.
